# Depletion of γ-glutamylcyclotransferase inhibits breast cancer cell growth via cellular senescence induction mediated by CDK inhibitor upregulation

**DOI:** 10.1186/s12885-016-2779-y

**Published:** 2016-09-22

**Authors:** Kengo Matsumura, Susumu Nakata, Keiko Taniguchi, Hiromi Ii, Eishi Ashihara, Susumu Kageyama, Akihiro Kawauchi, Tatsuhiro Yoshiki

**Affiliations:** 1 Department of Clinical Oncology, Kyoto Pharmaceutical University, Misasagi-Nakauchicho 5, Yamashinaku, Kyoto, 607-8414 Japan; 2 Department of Clinical and Translational Physiology, Kyoto Pharmaceutical University, Misasagi-Nakauchicho 5, Yamashinaku, Kyoto, 607-8414 Japan; 3 Department of Urology, Shiga University of Medical Science, Seta Tsukinowa-cho, Otsu, Shiga 520-2192 Japan

**Keywords:** γ-glutamylcyclotransferase, Cellular senescence, p21^WAF1/CIP1^, p16^INK4A^, Cell cycle arrest

## Abstract

**Background:**

Chromosome 7 open reading frame 24 (C7orf24) was originally identified as a highly expressed protein in various types of cancer, and later shown to be a γ-glutamylcyclotransferase (GGCT). GGCT depletion in cancer cells has anti-proliferative effects in vitro and in vivo, and it is therefore considered a promising candidate as a therapeutic target. However, the cellular events induced by GGCT depletion remain unclear.

**Methods:**

GGCT was depleted by siRNA in MCF7, MDA-MB-231, PC3, A172, Hela, and LNCaP cells. Induction of cellular senescence was evaluated with senescence-associated β-galactosidase (SA-β-Gal) staining. Expression levels of p21^WAF1/CIP1^ and p16^INK4A^ were assessed by qRT-PCR and Western blotting. Effects of simultaneous double knockdown of p21^WAF1/CIP1^ and p16^INK4A^ together with GGCT on cell cycle regulation and cell growth was measured by flow cytometry, and trypan blue dye exclusion test.

**Results:**

We found that GGCT knockdown induces significant cellular senescence in various cancer cells. Cyclin dependent kinase inhibitor p21^WAF1/CIP1^ and/or p16^INK4A^ were upregulated in all cell lines tested. Simultaneous knockdown of p21^WAF1/CIP1^ recovered the cell cycle arrest, attenuated cellular senescence induction, and rescued the subsequent growth inhibition in GGCT-silenced MCF7 breast cancer cells. In contrast, in GGCT silenced MDA-MB-231 breast cancer cells, GGCT depletion upregulated p16^INK4A^, which played a regulatory role in senescence induction, instead of p21^WAF1/CIP1^.

**Conclusions:**

Our findings demonstrate that induction of cellular senescence mediated by the upregulation of cyclin-dependent kinase inhibitors is a major event underlying the anti-proliferative effect of GGCT depletion in breast cancer cells, highlighting the potential of GGCT blockade as a therapeutic strategy to induce cellular senescence.

**Electronic supplementary material:**

The online version of this article (doi:10.1186/s12885-016-2779-y) contains supplementary material, which is available to authorized users.

## Background

Chromosome 7 open reading frame 24 (C7orf24) was first identified by proteomics analysis as a highly expressed protein in bladder cancer tissues by Kageyama et al. [1, 2] and later shown to be a γ-glutamylcyclotransferase (GGCT) [3]. In many cancers, GGCTs are expressed at higher levels in tumor than non-tumor tissues (58 % in cervical, 38 % in lung, 72 % in colon, and 46 % in breast cancer), and GGCT upregulation in tumor tissues is associated with poor clinical outcomes [4].

In previous in vitro studies, forced expression of GGCT in NIH3T3 cells (mouse fibroblasts cells) increased the rate of cell proliferation, and conversely, knockdown of GGCT significantly inhibited the growth of various cancer cells including lung, prostate, bladder, and breast cancer cells [2]. Regional injection [5] and systemic administration [6] of GGCT siRNA exerted anti-tumor effects in xenograft models in vivo. These findings suggest that GGCT is a promising candidate as a therapeutic target and diagnostic marker [7]. However, the cellular events associated with GGCT depletion have not been fully characterized to date, and the mechanisms underlying its inhibitory effect on the growth of cancer cells remain unknown.

Cellular senescence, which is characterized as permanent growth arrest of cells caused by limited cell division, was originally described by Hayflick [8]. Senescent cells undergo unique morphological changes and can become large, flat, and retractile [9], and they are characterized by increased SA-β-Gal activity [10]. Cellular senescence is induced by various stimuli including shortening telomeres, DNA damage, activated oncogenes, chromatin perturbation, and certain cancer therapeutics [9, 11].

The p53-p21^WAF1/CIP1^ and p16^INK4A^-pRb pathways mediate the induction of cellular senescence [11, 12]. Both p21^WAF1/CIP1^ and p16^INK4A^ are cyclin-dependent kinase (CDK) regulators [13, 14]. p21^WAF1/CIP1^ is a crucial transcriptional target of p53 and inhibits CDK2, causing cell cycle arrest at the G1 phase. p16^INK4A^ prevents the phosphorylation and inactivation of pRb, which forms a complex with E2F. The activity of E2F, a transcription factor that regulates the expression of genes associated with cell cycle progression, is suppressed when bound to pRb [15].

Cellular senescence affects not only normal cells but also cancer cells [16–18]. Chemotherapeutic agents induce cellular senescence in different cell lines, such as adriamycin in MCF7 and MDA-MB-231 cells [19, 20], camptothecin in HCT116 colon cancer cells [21, 22], and cisplatin in CNE1 nasopharyngeal carcinoma cells [23]. Recently, molecular targeted therapeutics against mutated BRAF were shown to exert anticancer effects via induction of cellular senescence [24]. In vivo studies showed that treatment with chemotherapeutic agents induces cellular senescence in HT1080 fibrosarcoma xenograft tumors treated with doxorubicin [25]. A clinical study reported that chemotherapeutic drugs increased the proportion of SA-β-Gal positive senescent cancer cells, and these senescent cells were frequently co-stained with p53 and p16^INK4A^ [26].

In the present study, we show that depletion of GGCT induces marked cellular senescence in various cancer cells. Knockdown of GGCT in MCF7 breast cancer cells significantly upregulated p21^WAF1/CIP1^, and the induction of cellular senescence and subsequent growth inhibition induced by GGCT depletion was dependent on p21^WAF1/CIP1^ upregulation. GGCT depletion induced the expression of p16^INK4A^, which regulated senescence induction in GGCT-silenced MDA-MB-231 breast cancer cells. To the best of our knowledge, this is the first study to identify cellular senescence induction mediated by the upregulation of CDK inhibitors as a major mechanism underlying the anti-proliferative effect of GGCT knockdown.

## Methods

### Cell lines and culture conditions

MCF7, MDA-MB-231, PC3, A172, Hela, and LNCaP cells were purchased from RIKEN BRC or ATCC and cultured in DMEM supplemented with 10 % fetal bovine serum (HyClone, South Logan, UT, USA) and 1 % penicillin and streptomycin. The cells were maintained in 5 % CO_2_ at 37 °C.

### Antibodies

Antibodies against various proteins were obtained from the following sources: mouse monoclonal antibodies, GGCT (6-1E, Cosmo Bio, Tokyo, Japan); p21^WAF1/CIP1^ (BD biosciences, New Jersey, NJ, USA); Caspase-8 (Cell Signaling Technology, Danvers, MA, USA); β-actin and GAPDH (Wako Pure Chemical Industries, Osaka, Japan); rabbit monoclonal antibodies, p16^INK4A^ (Abcam, Cambridge, MA, USA); PARP-1 (Enzo Life Science, Farmingdale, NY, USA); Caspase-3 (Cell Signaling Technology); and p53 (Cell Signaling Technology). Horse anti-mouse IgG-horseradish peroxidase (HRP) conjugates were from Vector Laboratories (Burlingame, CA, USA). Goat anti-rabbit IgG–HRP was from Jackson Immuno Research Laboratories (West Grove, PA, USA).

### Western blot analysis

Cell lysates were prepared with lysis buffer (50 mM Tris–HCl, 150 mM NaCl, 1 % NP-40, 0.5 % deoxycholate-Na, and 0.1 % SDS) supplemented with a protease inhibitor cocktail mix (Nacalai Tesque, Kyoto, Japan). Protein concentration was determined using the BCA protein assay (Bio-RAD, Hercules, CA, USA) according to the manufacturer’s protocol. Aliquots containing 20 μg of protein were separated by SDS-PAGE and transferred to PVDF membranes (Millipore, Billerica, MA, USA). After blocking with 5 % fat-free dry milk in phosphate buffered saline with 0.05 % Tween-20 (PBST), the membranes were incubated with primary and secondary antibodies in 3 % BSA in PBST. The proteins were visualized with the Clarity Western ECL Substrate (Bio-RAD). Chemiluminescence was detected by a cooled digital CCD camera, ChemiDoc XRS Plus (Bio-RAD).

### Cell cycle analysis

Cells were seeded in six-well plates and transfected with GGCT, p21^WAF1/CIP1^, p16^INK4A^, or non-targeting siRNA. The cells were washed and fixed with 70 % ethanol at −20 °C, and stained with propidium iodide (PI) at a concentration of 20 μg/ml in the presence of 200 μg/ml RNase A. DNA content was analyzed using FACSCalibur (BD Bioscience). At least 10,000 cells were analyzed for each sample.

### Trypan blue dye exclusion test

MCF7 and MDA-MB-231 cells were transfected with the indicated siRNAs 1 day after seeding. The standard trypan blue dye exclusion method was used with 0.4 % trypan blue solution (Wako).

### qRT-PCR analysis

Cells were lysed with Trizol (Thermo Fisher Scientific, Waltham, MA, USA), and total RNA extracts were purified with an RNeasy mini kit (Qiagen, Hilden, Germany) according to the manufacturer’s protocol. cDNA was synthesized by reverse transcription using the ReverTra Ace qPCR RT Master Mix (TOYOBO, Osaka, Japan). qRT-PCR analysis of cDNA was performed with THUNDERBIRD SYBR qPCR Mix (TOYOBO) according to the manufacturer’s protocol using the Light Cycler Nano System (Roche Diagnostics, Tokyo, Japan). Data were normalized to ARF1, a house-keeping gene. Primer sequences were listed in Additional file 1: Table S1 and S2.

### Transfection of siRNA targeting GGCT, p21^WAF1/CIP1^, and p16^INK4A^

Transient transfections were performed using Lipofectamine RNAi MAX (Invitrogen) according to the manufacturer’s protocol. Synthesized siRNAs were purchased from RNAi Co. LTD, Tokyo, or Gene Design Inc, Osaka, Japan. The siRNA sequences were listed in Additional file 1: Table S1 and S2. All siRNAs were transfected at a concentration of 10 nM. For double knockdown experiments, 20 nM of the non-targeting siRNA was transfected as a control.

### Staining of senescence-associated β-galactosidase

Cells were seeded in six-well plates, and transfected with siRNAs as described above. The cells were stained with SA-β-Gal 4 days after the transfection using the senescence kit (OZ Bioscience, San Diego, CA, USA) according to the manufacturer’s protocol. The cells were incubated with staining solution at 37 °C overnight, and SA-β-Gal positive cells were counted. For each evaluation, more than 400 cells were counted in at least 12 fields.

### Statistical analysis

All data were confirmed in at least three independent experiments. The data are expressed as the mean ± S.D., unless otherwise indicated. The two-tailed *Student’s t-test* was used for calculation of *p*-values using Excel software.

## Results

### Knockdown of GGCT suppresses the growth of MCF7 and MDA-MB-231 breast cancer cells

**Fig. 1:**
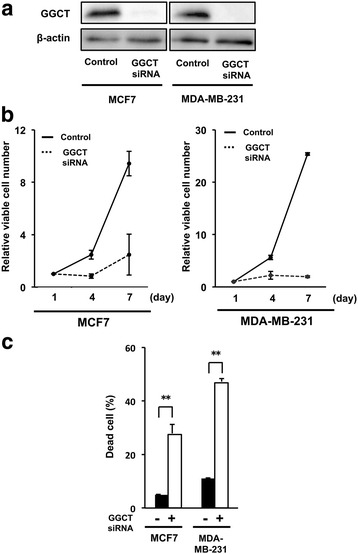
GGCT knockdown suppresses the growth of MCF7 and MDA-MB-231 cells. **a** MCF7 and MDA-MB-231 cells were transfected with siRNA targeting GGCT or non-target control siRNA, and the expression levels of GGCT in total cell lysates harvested 4 days after the transfection were analyzed by Western blotting. β-actin is shown as a loading control. **b** MCF7 and MDA-MB-231 cells were treated with GGCT siRNA or non-target siRNA. The relative number of trypan blue-negative viable cells at 1, 4, and 7 days after the transfection are shown. **c** Proportion of trypan blue-positive dead cells at 6 days after transfection. (** *p* < 0.01)

To study the mechanisms underlying the suppression of cell growth by GGCT knockdown, the efficiency of siRNA-mediated GGCT knockdown was first assessed by Western blotting in MCF7 and MDA-MB-231 breast cancer cells. The results showed a significant downregulation of GGCT in these cell lines (Fig. 1a). GGCT knockdown suppressed cell growth in MCF7 and MDA-MB-231 cells (Fig. 1b). The results of the trypan blue dye exclusion test revealed that GGCT knockdown significantly increased the proportion of dead cells positively stained with trypan blue in both MCF7 and MDA-MB-231 cell lines (Fig. 1c).

### Cellular senescence was induced by GGCT knockdown in various cancer cells

**Fig. 2:**
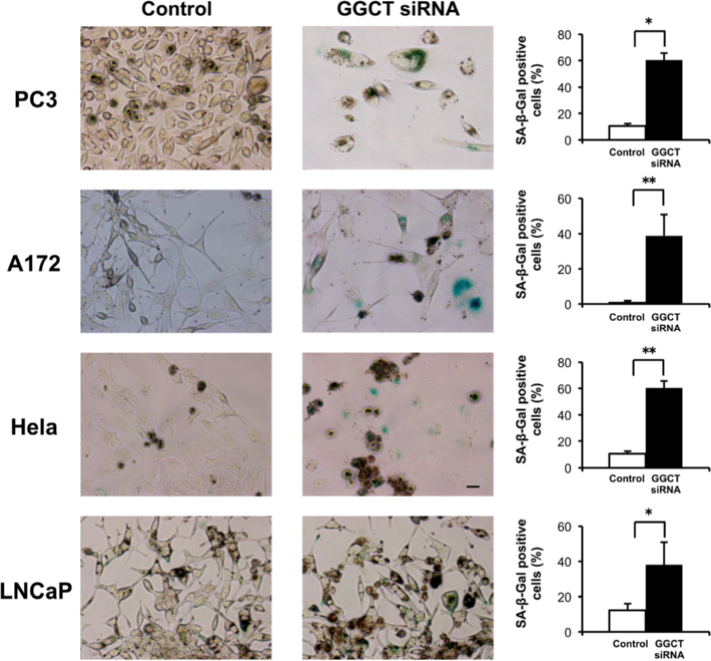
Depletion of GGCT induces cellular senescence in various cancer cells. The indicated cancer cell lines were transfected with siRNA targeting GGCT or non-target control siRNA, and cellular senescence was evaluated by SA-β-Gal staining at 4 days after transfection. Representative images and proportion of SA-β-Gal positive cells are shown. Scale bar represents 50 μm

GGCT-depleted cells exhibited a pronounced flat and enlarged morphology, a characteristic phenotypic change associated with cellular senescence. Cells were then stained with SA-β-Gal, a specific marker for senescent cells [10]. As shown in Fig. 2, knockdown of GGCT induced cellular senescence, as detected by SA-β-Gal staining, in MCF7 and MDA-MB-231 cells, as well as other cancer cell lines, including PC3 and LNCaP prostate cancer cells, HeLa cervical cancer cells, and A172 glioblastoma cells.

### Cellular senescence induced by GGCT knockdown was dependent on p21^WAF1/CIP1^ upregulation in MCF7 cells

**Fig. 3:**
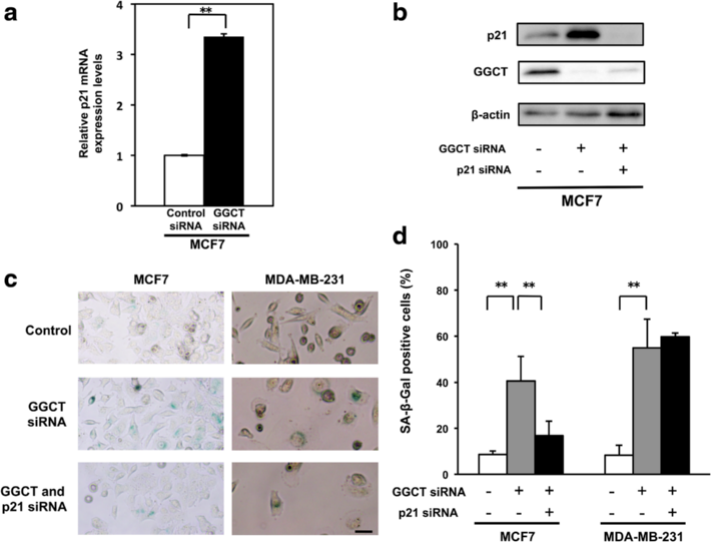
Induction of senescence by GGCT knockdown depends on p21^WAF1/CIP1^ upregulation in MCF7 cells. **a**-**b** The expression levels of p21^WAF1/CIP1^ (**a**) mRNA and (**b**) protein in MCF7 cells harvested 4 days after transfection with the indicated siRNAs were analyzed by qRT-PCR and western blotting, respectively. **c** Representative images of SA-β-Gal staining 4 days after transfection with the indicated siRNAs in MCF7 and MDA231-MB cells. Scale bar represents 50 μm. **d** The numbers of SA-β-Gal positive cells were counted and their ratios in total cells are shown. (^**^ *p* < 0.01)

Since p21^WAF1/CIP1^ is an important regulator of cellular senescence [11, 17, 27, 28], we investigated the effect of GGCT knockdown on the expression of p21^WAF1/CIP1^. Quantitative real-time PCR and Western blot analysis showed a significant induction of p21^WAF1/CIP1^ expression by GGCT knockdown in MCF7 cells (Fig. 3a and b). We confirmed that expression levels of p21^WAF1/CIP1^ protein by GGCT knockdown were also upregulated in PC3, A172, and Hela cells (Additional file 2: Figure S1). To determine whether p21^WAF1/CIP1^ plays a role in the induction of cellular senescence, the proportion of SA-β-Gal positive cells was measured in MCF7 cells treated with p21^WAF1/CIP1^ targeting siRNA together with GGCT siRNA. The double knockdown of p21^WAF1/CIP1^ and GGCT efficiently suppressed both GGCT and p21^WAF1/CIP1^ proteins (Fig. 3b), and resulted in a significant decrease in the number of SA-β-Gal positive cells compared with that in cells treated with GGCT siRNA alone (Fig. 3c and d). However, no significant changes in the proportion of senescent cells were observed in MDA-MB-231 cells (Fig. 3d), consistent with the very low expression levels of p21 and absence of significant induction of p21 by GGCT knockdown (Additional file 2: Figure S1).

### p21^WAF1/CIP1^-dependent G0/G1 arrest is critical for growth inhibition induced by knockdown of GGCT in MCF7 cells

**Fig. 4:**
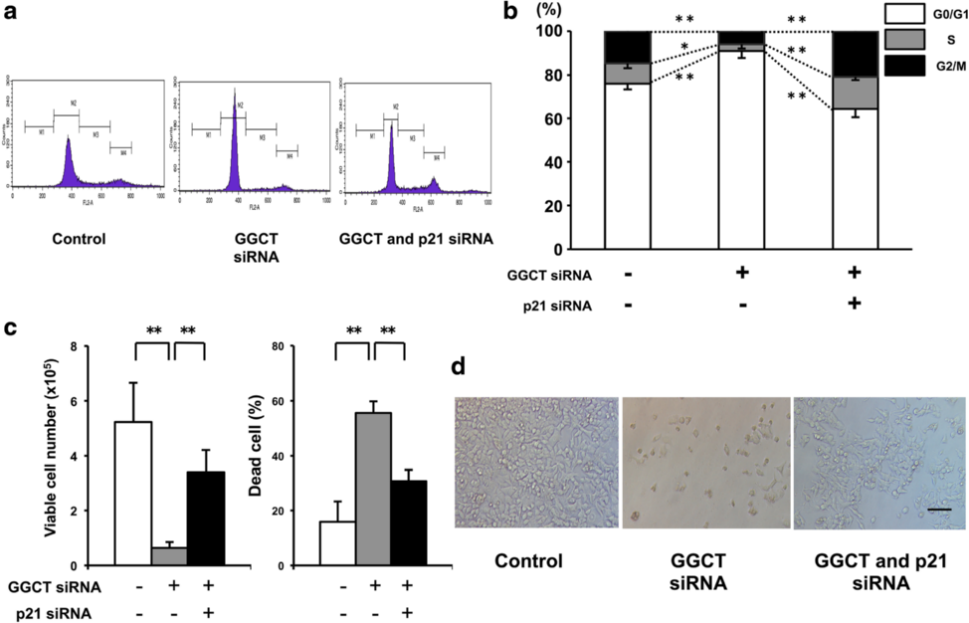
Upregulation of p21^WAF1/CIP1^ is critical for G0/G1 arrest and consequent growth inhibition by GGCT knockdown. **a**-**b** The distribution of cell cycle phases in MCF7 cells was analyzed by flow cytometry 4 days after transfection with the indicated siRNAs. Representative histograms (**a**) and quantified distributions are shown (**b**). **c**-**d** The number of Trypan blue-negative viable MCF cells (**c**) and the proportion of dead cells (**d**) at 7 days after transfection with the indicated siRNAs are shown. (^*^ *p* < 0.05, ^**^ *p* < 0.01)

Next, we investigated the effect of knockdown of GGCT alone or together with p21^WAF1/CIP1^ on cell cycle progression. Transfection of MCF7 cells with GGCT siRNA led to a significant increase in the percentage of G0/G1 phase cells (from 74.7 to 90.4 %), and a significant decrease in S and G2/M phase cells (from 9.3 to 3.0 % and from 14.4 to 6.1 % respectively, Fig. 4a and b), whereas no induction of the sub-G1 population was observed (Fig. 4a). Apoptotic signaling molecules, such as caspases and PARP, were not activated by GGCT knockdown (Additional file 3: Figure S2), indicating that the cell death induced after cellular senescence was mediated by a mechanism distinct from apoptosis. The concurrent knockdown of p21^WAF1/CIP1^ and GGCT led to a shift in the percentages of G0/G1, G2/M, and S phase cells (Fig. 4b), demonstrating the important role of p21^WAF1/CIP1^ induction in cell cycle regulation. However, no significant recovery of cell cycle distribution by p21^WAF1/CIP1^ knockdown was observed in GGCT-silenced MDA-MB-231 cells (data not shown). The effect of p21^WAF1/CIP1^ on growth inhibition induced by GGCT knockdown was assessed in MCF7 cells. The double knockdown of p21^WAF1/CIP1^ and GGCT led to a significant recovery of viable cell numbers in MCF7 cells (Fig. 4c and d). Moreover, knockdown of p21^WAF1/CIP1^ significantly recovered the dead cell ratio, when compared with that observed after knockdown of GGCT alone (Fig. 4c, right panel).

### Upregulated p16^INK4A^ plays a role in cellular senescence in GGCT-depleted MDA-MB-231 cells, but not in MCF7 cells

**Fig. 5:**
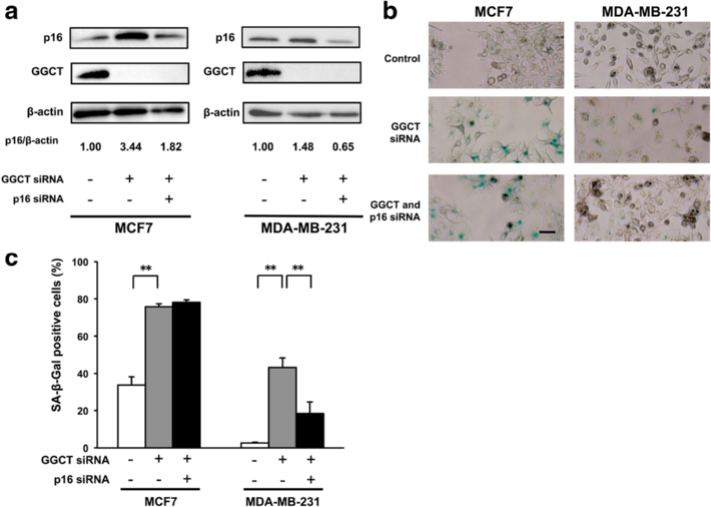
p16^INK4A^ regulates GGCT knockdown-induced senescence in MDA-MB-231 cells. **a** The expression levels of p16^INK4A^ in MCF7 and MDA-MB-231 cells harvested 4 days after transfection with the indicated siRNAs were analyzed by western blotting. β-actin is shown as loading controls. **b** Representative images of SA-β-Gal staining 4 days after transfection with the indicated siRNAs in MCF7 and MDA231-MB cells. Scale bar represents 50 μm. **c** The number of SA-β-Gal positive cells was counted and their ratios in total cells are shown. (^**^ *p* < 0.01)

p21^WAF1/CIP1^ played a critical role in the inhibition of MCF7 cell growth induced by GGCT knockdown; however, p21^WAF1/CIP1^ was not induced in senescent MDA-MB-231 cells after efficient knockdown of GGCT (Additional file 2: Figure S1). To identify the mediator of senescence induction in MDA-MB-231 cells, the expression levels of p16^INK4A^, an important regulator of senescence [11, 28, 29], were assessed. As shown in Fig. 5a, knockdown of GGCT significantly induced p16^INK4A^ expression in both MCF7 and MDA-MB-231 cells. We also confirmed that expression levels of p16^INK4A^ protein were also induced in PC3, Hela, and LNCaP cells (Additional file 2: Figure S1). Knockdown of p16^INK4A^ together with GGCT siRNA significantly downregulated p16^INK4A^ to control levels (Fig. 5a). Knockdown of p16^INK4A^ had no impact on senescence induction in MCF7 cells, consistent with the critical contribution of p21^WAF1/CIP1^. By contrast, p16^INK4A^ knockdown reduced the SA-β-Gal positive senescent population in MDA-MB-231 cells (Fig. 5b and c).

### Induction of p16^INK4A^ plays a role in growth inhibition caused by GGCT knockdown in MDA-MB-231 cells

**Fig. 6:**
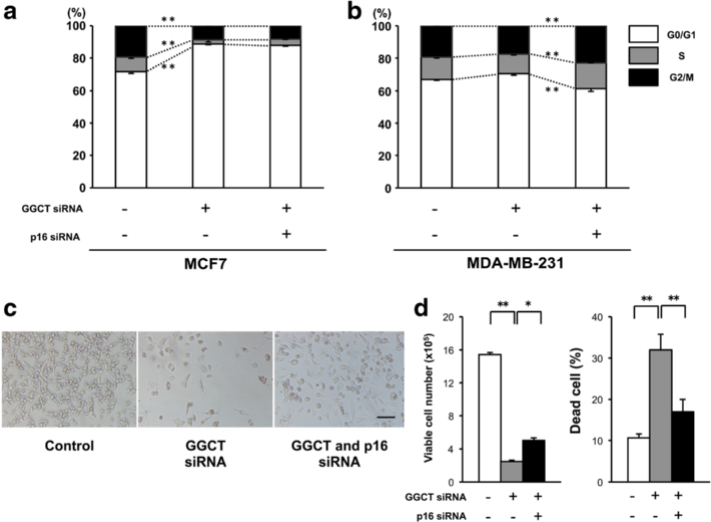
Blockage of p16^INK4A^ induction attenuates cell cycle arrest and growth inhibition caused by GGCT knockdown. **a**-**b** The distribution of cell cycle phases in MCF7 (A) and MDA-MB-231 (**b**) cells was analyzed by flow cytometry 4 days after transfection with the siRNAs indicated. **c**-**d** Representative images of MDA-MB-231 cells (**c**), the number of Trypan blue-negative viable MDA-MB-231 cells (**d**, left panel), and the proportion of dead cells (**d**, right panel) at 7 days after transfection with the indicated siRNAs are shown. (^*^ *p* < 0.05, ^**^ *p* < 0.01)

Next, we investigated the effect of modulation of p16^INK4A^ levels on cell cycle regulation in GGCT-depleted cells. MCF7 cells showed no recovery of cell cycle status by p16^INK4A^ blockade (Fig. 6a), whereas MDA-MB-231 cells showed a slight but significant recovery of the G0/G1 arrest and increase of S and G2/M phase by the p16^INK4A^ blockade (Fig. 6b). The effect of p16^INK4A^ depletion on growth inhibition induced by GGCT knockdown was assessed in MDA-MB-231 cells. As shown in Fig. 6c and d, concomitant knockdown of p16^INK4A^ significantly recovered the inhibition of MDA-MB-231 cell growth caused by GGCT silencing. Moreover, double knockdown decreased the proportion of dead cells (Fig. 6d right panel).

## Discussion

A growing body of evidence indicates that GGCT is a promising candidate as a therapeutic target and biomarker in cancer [7]. To date, however, the mechanisms underlying the inhibition of cancer cell growth induced by GGCT knockdown remain unknown. Here, we show that depletion of GGCT induces cellular senescence in different cancer cells, inhibits cell growth, and promotes cell death.

The temporal dynamics of phenotypic changes induced by GGCT knockdown were unique. GGCT-silenced cells proliferated slowly for 4 days and showed no evidence of cell death induction, despite undergoing morphological changes resulting in flat and enlarged shapes. Thereafter, from 6 to 7 days post transfection, the cells started to die, without evidence of nuclear fragmentation or activation of apoptotic pathways. GGCT silencing caused cellular senescence not only in MCF7 and MDA-MB-231 breast cancer cells, but also in other cancer cells, as detected by SA-β-Gal positivity. This suggested that the induction of senescence is a universal phenomenon upon GGCT depletion.

Cellular senescence is defined as permanent cell cycle arrest, and senescent cells lose proliferative potency [16, 17]. Various molecular mechanisms of cellular senescence have been proposed [9, 11, 28]. Among them, the p21^WAF1/CIP1^ mediated induction of cellular senescence was shown in various cellular contexts [11]. Microarray-based studies suggest that p21^WAF1/CIP1^ expression positively correlates with the induction of genes associated with senescence [30]. Johmura et al. reported that p21^WAF1/CIP1^ is upregulated at the early stages of cellular senescence [12]. Our results also showed that p21^WAF1/CIP1^ was upregulated in GGCT-silenced MCF7 cells at the mRNA and protein levels (Fig. 3a and b). In parallel with the upregulation of p21^WAF1/CIP1^, GGCT-depleted MCF7 cells stained positive for SA-β-Gal (Fig. 3c), indicating the induction of cellular senescence. The induction of cellular senescence was dependent on the upregulation of p21^WAF1/CIP1^ in MCF7 cells, as demonstrated by the effect of simultaneous depletion of GGCT and p21^WAF1/CIP1^ (Fig. 3d).

In addition to its role as an effector of cellular senescence, p21^WAF1/CIP1^ has many functions, including cell cycle regulation and the modulation of apoptotic pathways [27, 31]. As a member of the Cip and Kip family of CDK inhibitors [27], p21^WAF1/CIP1^ is a key regulator of cell cycle checkpoints at G1 and S phases [27, 31]. Consistent with this, the marked induction of p21^WAF1/CIP1^ upon GGCT silencing resulted in cell cycle arrest at the G0/G1 phase in MCF7 cells (Fig. 4a). Our data using double knockdown clearly demonstrate that the induction of G0/G1 arrest occurred in a p21^WAF1/CIP1^-dependent manner (Fig. 4c and d).

p53 is a key transactivator of p21^WAF1/CIP1^ [32]. Although MCF7 cells have wild-type p53 [21], GGCT knockdown did not induce p53 in MCF7 and MDA-MB-231 cells (Additional file 4: Figure S3), suggesting that p21^WAF1/CIP1^ upregulation was mediated by p53-independent pathways in MCF7 cells. In addition, p21^WAF1/CIP1^ was significantly upregulated in PC3 cells (Additional file 2: Figure S1), which harbor a null p53 mutant, suggesting that p21^WAF1/CIP1^ induction by GGCT depletion was mediated by p53-independent mechanisms. Supporting these findings, multiple p53-independent mechanisms of transactivation of p21^WAF1/CIP1^ are known [29]. Further investigations are needed to clarify the precise mechanisms underlying the upregulation of p21^WAF1/CIP1^.

The attenuation of the G0/G1 cell cycle arrest and subsequent cellular senescence by simultaneous knockdown of p21^WAF1/CIP1^ resulted in significant recovery of cell growth and decrease of dead cell population in GGCT-depleted MCF7 cells. These results indicate that the induction of cellular senescence due to the permanent loss of proliferative potential led to cell death induction, highlighting p21^WAF1/CIP1^ as an important mediator of GGCT knockdown-mediated growth suppression in MCF7 cells.

In contrast to the upregulation of p21^WAF1/CIP1^ in MCF7 cells, depletion of GGCT induced the upregulation of p16^INK4A^ (Fig. 5a) in MDA-MB-231 cells, but not that of p21^WAF1/CIP1^. p16^INK4A^, a CDK inhibitor, is a p53-independent regulator of oncogene-induced cell cycle arrest and senescence [29, 32]. We showed that blocking p16^INK4A^ upregulation, at least in part, attenuated the induction of cellular senescence and subsequent suppression of cell growth accompanied by a massive induction of cell death. These results suggest that p16^INK4A^ upregulation mediated cellular senescence leading to the inhibition of cell growth in GGCT-silenced MDA-MB-231 cells. However, the simultaneous knockdown of p16^INK4A^ could not recover cellular senescence as efficiently as in the case of p21^WAF1/CIP1^ in MCF7 cells, suggesting that other molecules may play a role in the induction of senescence by GGCT depletion in MDA-MB-231 cells (Fig. 5b and c).

## Conclusions

Taken together, our results indicate that depletion of GGCT induces CDK inhibitor(s) that mediate cell cycle arrest and subsequent cellular senescence in various cancer cells, leading to the significant suppression of cell growth. The CDK inhibitor(s) induced by GGCT depletion may vary according to cell type. Our results shed new light on the mechanisms underlying the anticancer effects of GGCT-targeting and highlight the potential of GGCT blockade as a therapeutic strategy to induce cellular senescence in cancer cells.

## Additional files

Additional file 1: Table S1 and Table S2. Represent genomic PCR primer sequences and small interfering RNA sequences respectively. (DOCX 15 kb) Additional file 2: Figure S1. Knockdown of GGCT upregulates p21^WAF1/CIP1^ and/or p16^INK4A^ in various cancer cells. Expression levels of p21^WAF1/CIP1^ and p16^INK4A^ protein in GGCT-depleted various cancer cells indicated were analyzed by Western blotting. (PDF 2326 kb) Additional file 3: Figure S2. Apoptosis is not involved in cell death induced by GGCT knockdown MCF7 cells. The expression levels of PARP, caspase-3, and caspase-8 in MCF7 cells harvested 6 days after transfection with the indicated siRNAs indicated were analyzed by western blotting. β-actin is shown as the loading control. One μM Etoposide (VP-16) treated HL60 cells were used as positive controls to detect the cleaved forms of PARP and caspases. (PDF 3526 kb) Additional file 4: Figure S3. Knockdown of GGCT does not induce p53 in MCF7 and MDA-MB-231 cells. Expression levels of p53 protein in GGCT-depleted MCF7 and MDA-MB-231 cells were analyzed by Western blotting. (PDF 1540 kb) Additional file 5: Figure S4. Evaluation cellular senescence on day 7 after the transfection of GGCT siRNA. Cellular senescence was evaluated by SA-β-Gal staining at 7 days after transfection in MCF7 and MDA-MB-231 cells. Representative images and proportion of SA-β-Gal positive cells are shown. Scale bar represents 50 μm. (PDF 12523 kb)

## Abbreviations

ARF1: ADP-ribosylation factor 1
C7orf24: Human chromosome 7 orf 24
CDK: Cyclin-dependent kinase
GAPDH: Glyceraldehyde-phosphate dehydrogenase
GGCT: γ-glutamylcyclotransferase
pRb: Protein retinoblastoma
qRT-PCR: Quantitative real-time polymerase chain reaction
RNAi: RNA interference
SA-β-Gal: Senescence-associated β − galactosidase
siRNA: Small interfering RNA
