# Supplementary material for: Depletion of γ-glutamylcyclotransferase inhibits breast cancer cell growth via cellular senescence induction mediated by CDK inhibitor upregulation
Source: BMC Cancer. 2016 Sep 22;16:748. doi: 10.1186/s12885-016-2779-y (PMC5034417; doi:10.1186/s12885-016-2779-y)
Supplement: Additional file 1: Table S1 and Table S2. — Represent genomic PCR primer sequences and small interfering RNA sequences respectively. (DOCX 15 kb) [file 12885_2016_2779_MOESM1_ESM.docx]

| **Target gene** | **Forward** | **Reverse** |
| --- | --- | --- |
| p21^WAF1/CIP1^ | CGATGGAACTTCGACTTTGTCA | GCACAAGGGTACAAGACAGTG |
| ARF1 | GACCACGATCCTCTACAAGC | TCCCACACAGTGAAGCTGATG |

**Additional file 1**

**Table S1: Genomic PCR primer sequences**

**Table S2: Small interfering RNA sequences**

| **Target gene** | **Sense(5’→3’)** | **Anti-sense(5’→3’)** |
| --- | --- | --- |
| Non targeting | GUACCGCACGUCAUUCGUAUC | UACGAAUGACGUGCGGUACGU |
| GGCT | UGACUAUACAGGAAAGGUCTT | GACCUUUCCUGUAUAGUCATT |
| p21^WAF1/CIP1^ | CUGUACUGUUCUGUGUCUU | AAGACACAGAACAGUACAG |
| p16^INK4A^ | CGCACCGAAUAGUUACGGUTT | ACCGUAACUAUUCGGUGCGTT |
