# Supplementary figures and images for: Depletion of γ-glutamylcyclotransferase inhibits breast cancer cell growth via cellular senescence induction mediated by CDK inhibitor upregulation
Source: BMC Cancer. 2016 Sep 22;16:748. doi: 10.1186/s12885-016-2779-y (PMC5034417; doi:10.1186/s12885-016-2779-y)

**Supplementary Figure 1**

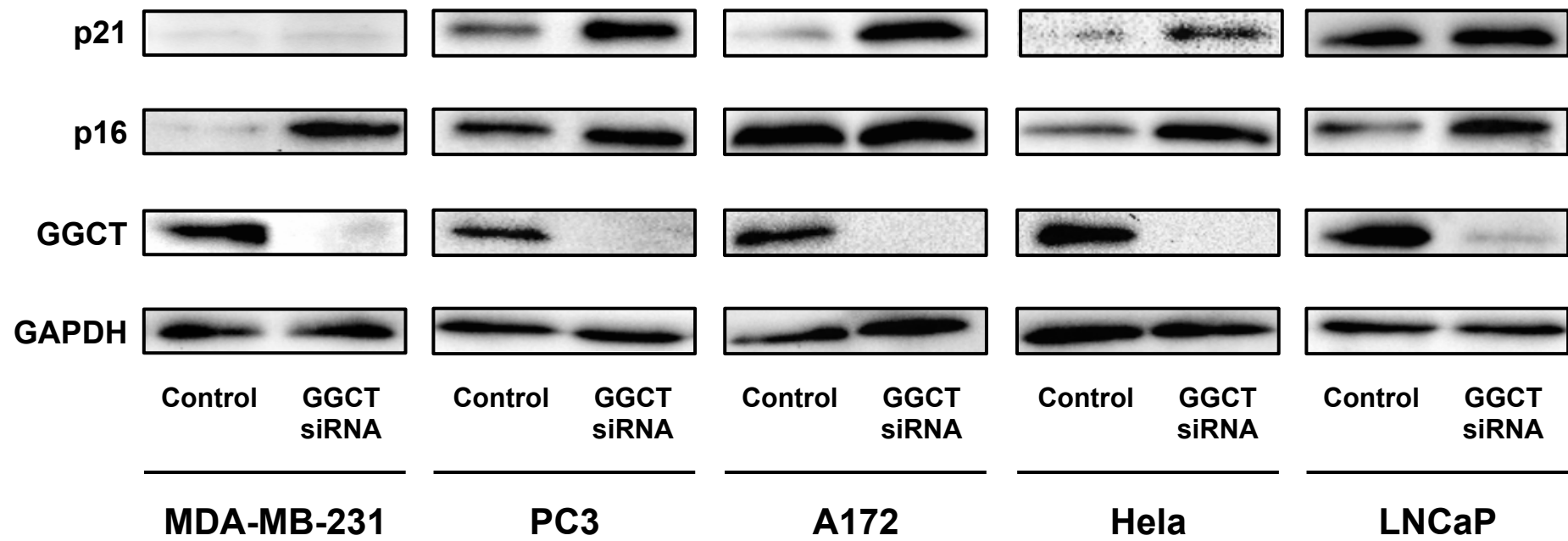

Supplement: Additional file 2: Figure S1. — Knockdown of GGCT upregulates p21WAF1/CIP1 and/or p16INK4A in various cancer cells. Expression levels of p21WAF1/CIP1 and p16INK4A protein in GGCT-depleted various cancer cells indicated were analyzed by Western blotting. (PDF 2326 kb) [file 12885_2016_2779_MOESM2_ESM.pdf]

Supplementary Figure 2

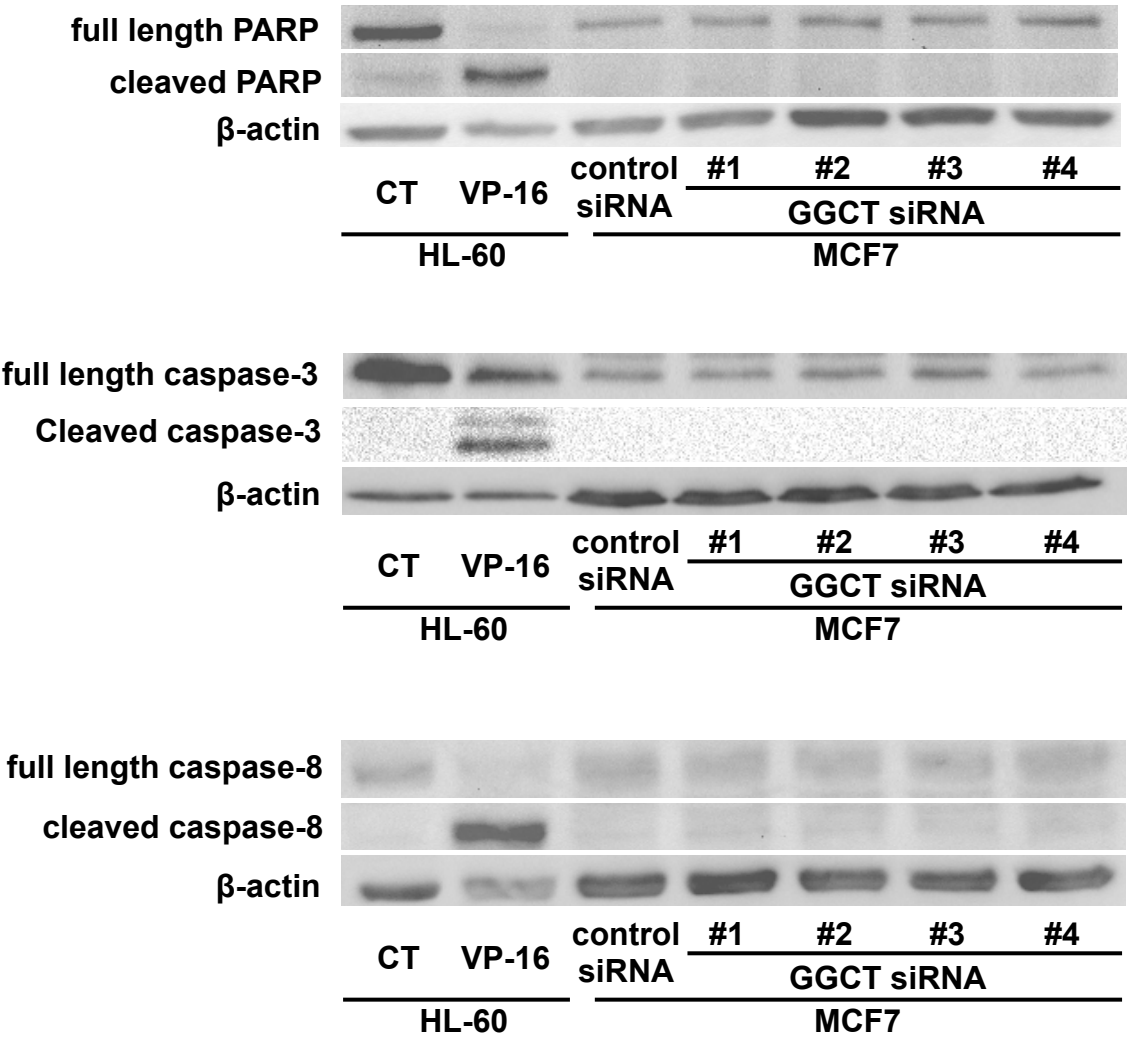

Supplement: Additional file 3: Figure S2. — Apoptosis is not involved in cell death induced by GGCT knockdown MCF7 cells. The expression levels of PARP, caspase-3, and caspase-8 in MCF7 cells harvested 6 days after transfection with the indicated siRNAs indicated were analyzed by western blotting. β-actin is shown as the loading control. One μM Etoposide (VP-16) treated HL60 cells were used as positive controls to detect the cleaved forms of PARP and caspases. (PDF 3526 kb) [file 12885_2016_2779_MOESM3_ESM.pdf]

### Supplementary Figure 3

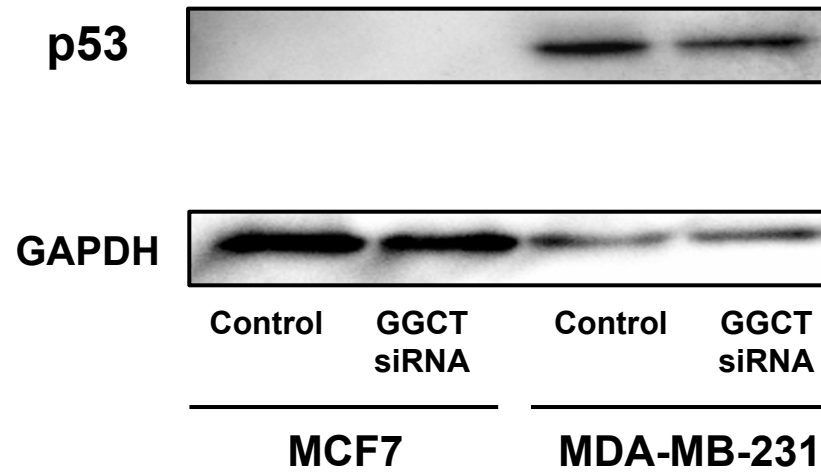

Supplement: Additional file 4: Figure S3. — Knockdown of GGCT does not induce p53 in MCF7 and MDA-MB-231 cells. Expression levels of p53 protein in GGCT-depleted MCF7 and MDA-MB-231 cells were analyzed by Western blotting. (PDF 1540 kb) [file 12885_2016_2779_MOESM4_ESM.pdf]

## Supplementary Figure 4

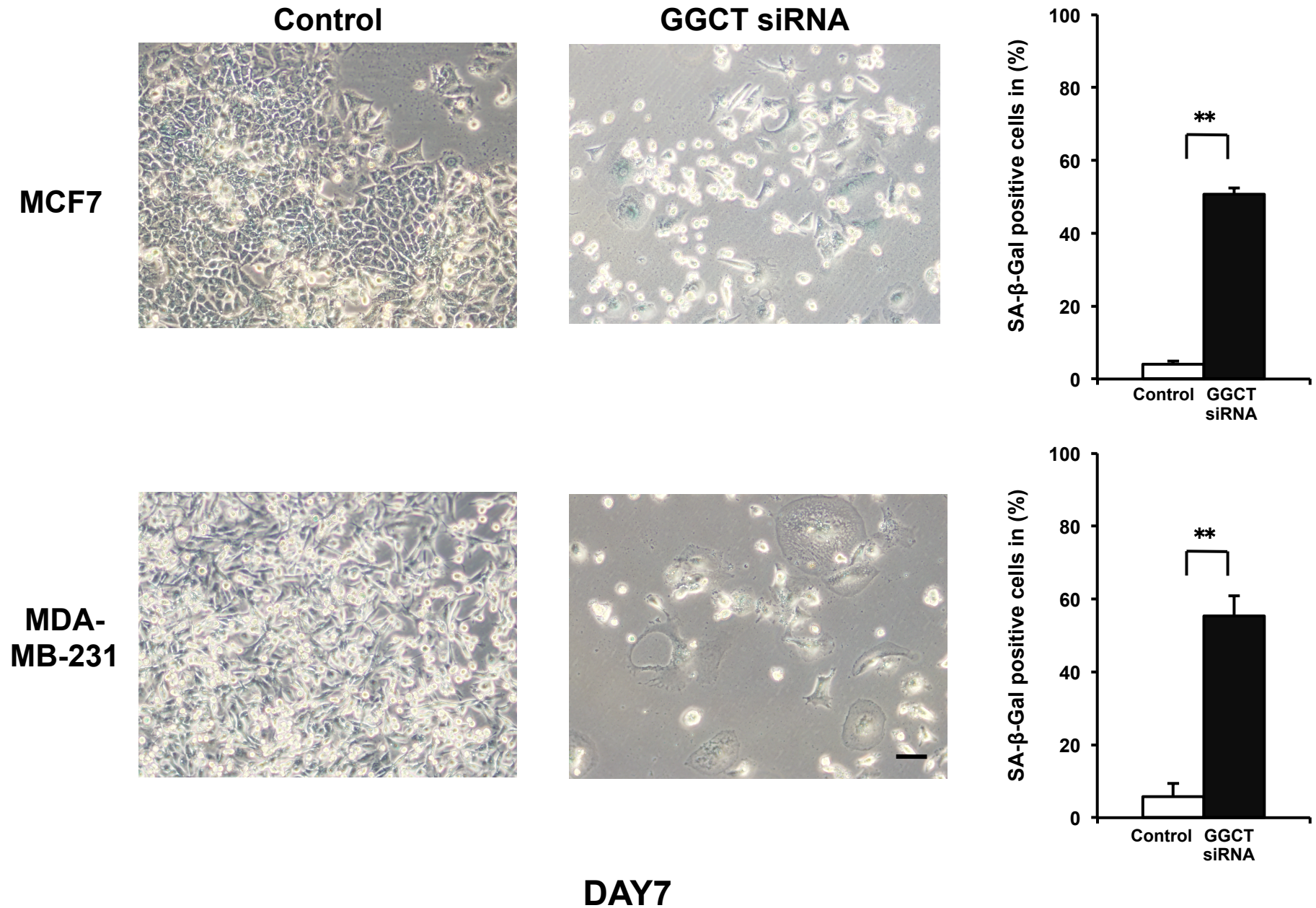

Supplement: Additional file 5: Figure S4. — Evaluation cellular senescence on day 7 after the transfection of GGCT siRNA. Cellular senescence was evaluated by SA-β-Gal staining at 7 days after transfection in MCF7 and MDA-MB-231 cells. Representative images and proportion of SA-β-Gal positive cells are shown. Scale bar represents 50 μm. (PDF 12523 kb) [file 12885_2016_2779_MOESM5_ESM.pdf]
